# The impact of remdesivir on SARS-CoV-2 evolution in vivo

**DOI:** 10.1172/jci.insight.182376

**Published:** 2025-01-21

**Authors:** Ted Ling-Hu, Lacy M. Simons, Estefany Rios-Guzman, Alexandre Machado Carvalho, Maria Francesca R. Agnes, Arghavan Alisoltanidehkordi, Egon A. Ozer, Ramon Lorenzo-Redondo, Judd F. Hultquist

**Affiliations:** 1 Division of Infectious Diseases, Northwestern University Feinberg School of Medicine, Chicago, Illinois, USA.; 2 Center for Pathogen Genomics and Microbial Evolution, Northwestern University Havey Institute for Global Health, Chicago, Illinois, USA.

**Keywords:** COVID-19, Therapeutics, Drug therapy

## Abstract

The impact of remdesivir on SARS-CoV-2 diversity and evolution in vivo has remained unclear. In this single-center, retrospective cohort study, we assessed SARS-CoV-2 diversification and diversity over time in a cohort of hospitalized patients who did or did not receive remdesivir. Whole-genome sequencing was performed on 98 paired specimens collected from 49 patients before and after remdesivir administration. The genetic divergence between paired specimens was not significantly different in this cohort compared with that in a control group of patients who did not receive the drug. However, when we focused on minority variants, several positions showed preferential diversification after remdesivir treatment, some of which were associated with specific variants of concern. Most notably, remdesivir administration resulted in strong selection for a nonsynonymous mutation in nsp12, G671S, previously associated with enhanced viral fitness. This same mutation was found to be enriched in a second cohort of 143 inpatients with specimens collected after remdesivir administration compared with controls. Only one other mutation previously implicated in remdesivir resistance (nsp12:V792I) was found to be preferentially selected for after remdesivir administration. These data suggest that SARS-CoV-2 variants with enhanced replicative fitness may be selected for in the presence of antiviral therapy as an indirect means to overcome this selective pressure.

## Abstract 2

This study looked at how remdesivir affects the diversity and changes in SARS-CoV-2 over time. It found that while overall genetic differences weren’t significant, specific mutations linked to stronger viruses increased after remdesivir treatment.

## Introduction

Since its emergence in 2019, SARS-CoV-2 has become a leading cause of respiratory illness worldwide (1–4). Despite high levels of natural and vaccine-based immunity in the population, individuals in high-risk groups — including the elderly and immunocompromised — may still experience severe disease (5–7). To treat severe COVID-19, physicians rely on an increasing number of direct-acting antivirals (DAAs) to reduce the risk of hospitalization and death (8, 9). These currently include inhibitors of the viral RNA-dependent RNA polymerase (RdRp, encoded by nonstructural protein 12 [nsp12]) (10, 11) and the major protease (mPro, encoded by nsp5) (12), though other drug targets are being actively explored.

Remdesivir was the first FDA-approved antiviral for the treatment of COVID-19 in the United States (13). Remdesivir is a phosphoramidate prodrug that is metabolized in the body to the active form of remdesivir triphosphate (RTP) (14). RTP is incorporated into viral RNA by RdRp, stalling downstream transcription (14). Remdesivir was originally developed for the treatment of Ebola virus, but drug-repurposing efforts early in the COVID-19 pandemic demonstrated its antiviral activity against SARS-CoV-2 in cell lines and mouse models (15, 16). Several clinical trials (including the ACTT-1 and PINETREE trials, among others) have since demonstrated that remdesivir reduces hospitalization and death for at-risk groups as compared with placebo (17, 18). However, several clinical trials of remdesivir have also failed to reach their clinical end points, perhaps most notably the SOLIDARITY trial conducted by the WHO, which showed no significant effect of remdesivir on patients who were already hospitalized as measured by overall mortality rate, initiation of ventilation, and duration of hospital stay (19–21). Several factors have been suggested to account for these different outcomes, including differences in the study population, drug administration criteria, and clinical end points (22, 23). Despite some ambiguity in clinical efficacy, remdesivir is still among the most widely administered DAA for the treatment of COVID-19 in hospitalized patients. The standard treatment regimen for inpatient adults not requiring intensive care (i.e., invasive mechanical ventilation or supplemental oxygen) is a 5-day course administered intravenously or until hospital discharge, though the time course may be altered according to severity (24).

The ability of SARS-CoV-2 to diversify within a host, particularly in cases of persistent infection of immunosuppressed or immunocompromised hosts, raises the possibility that the virus may evolve antiviral resistance to remdesivir (as has been observed with the evolution of resistance to the Mpro inhibitor nirmatrelvir) (25–27). Several in vitro studies have successfully selected for remdesivir-resistant viruses in cell culture, though these have been largely observed to have lower overall fitness (28). Indeed, the resistance mutations observed in vitro have been only rarely observed in clinical isolates. While some patient studies have looked for the evolution of remdesivir resistance in vivo, these have generally been limited to case studies or small patient cohorts, have lacked matched control cohorts that did not receive remdesivir, and have identified only a few putative resistance mutations (29, 30). Overall, the effect of remdesivir on SARS-CoV-2 diversification and evolution in vivo has, to our knowledge, yet to be studied in a large patient cohort with matched controls.

Here, we present a single-center retrospective cohort study examining the effect of remdesivir administration on SARS-CoV-2 diversification and evolution in adult patients diagnosed with COVID-19. SARS-CoV-2 whole-genome sequence data from 192 adult inpatients who were administered remdesivir was compared with those from 193 adult inpatient controls who did not receive remdesivir. Longitudinal specimens were used to model positional diversity and were assessed for the emergence of previously reported resistance mutations in nsp12. Several positions across the genome were found to diversify following remdesivir administration, though these were largely outside of nsp12 and included only 2 previously identified resistance mutations, nsp12:G671S and nsp12:V792I. Nearly half of the mutations selected for after remdesivir treatment emerged in different variants of concern (VOCs), several of which have been previously associated with enhanced viral fitness. Overall, these data suggest that while remdesivir treatment may not select for resistance mutations in nsp12 that directly reduce antiviral effectiveness, it does induce a selective pressure for the evolution of a virus with overall enhanced fitness.

## Results

### Most mutations previously implicated in remdesivir resistance are low-frequency.

Several studies have been conducted to identify potential remdesivir resistance mutations in SARS-CoV-2 using a variety of approaches, including sequencing of clinical isolates following remdesivir administration, selection of resistant viruses in cell culture, and computational modeling. We first conducted a literature review to identify mutations in nsp12, which was previously implicated in remdesivir resistance. This review identified 29 unique mutations in nsp12 across 19 peer-reviewed publications (Supplemental Table 1; supplemental material available online with this article; https://doi.org/10.1172/jci.insight.182376DS1). A majority of these studies were conducted by serial passaging of SARS-CoV-2 in the presence or absence of remdesivir and subsequent sequencing of outgrowth cultures (i.e., by in vitro methodologies) (28, 31–36). A smaller subset of studies looked for the emergence of remdesivir resistance mutations in patients with COVID-19 following treatment, usually in immunocompromised or immunosuppressed hosts with persistent infection (i.e., by sequencing of in vivo specimens) (37, 38). Unfortunately, these studies have largely been limited to single-case studies or small-cohort studies lacking control groups that did not receive remdesivir (29, 30, 39–44). Last, several published studies have relied purely on computational methods to predict potential resistance mutations based on remdesivir’s mechanism of action, the SARS-CoV-2 RdRp structure, and mutations that have been reported in existing public repositories (i.e., by in silico methodologies) (45–48).

**Figure 1:**
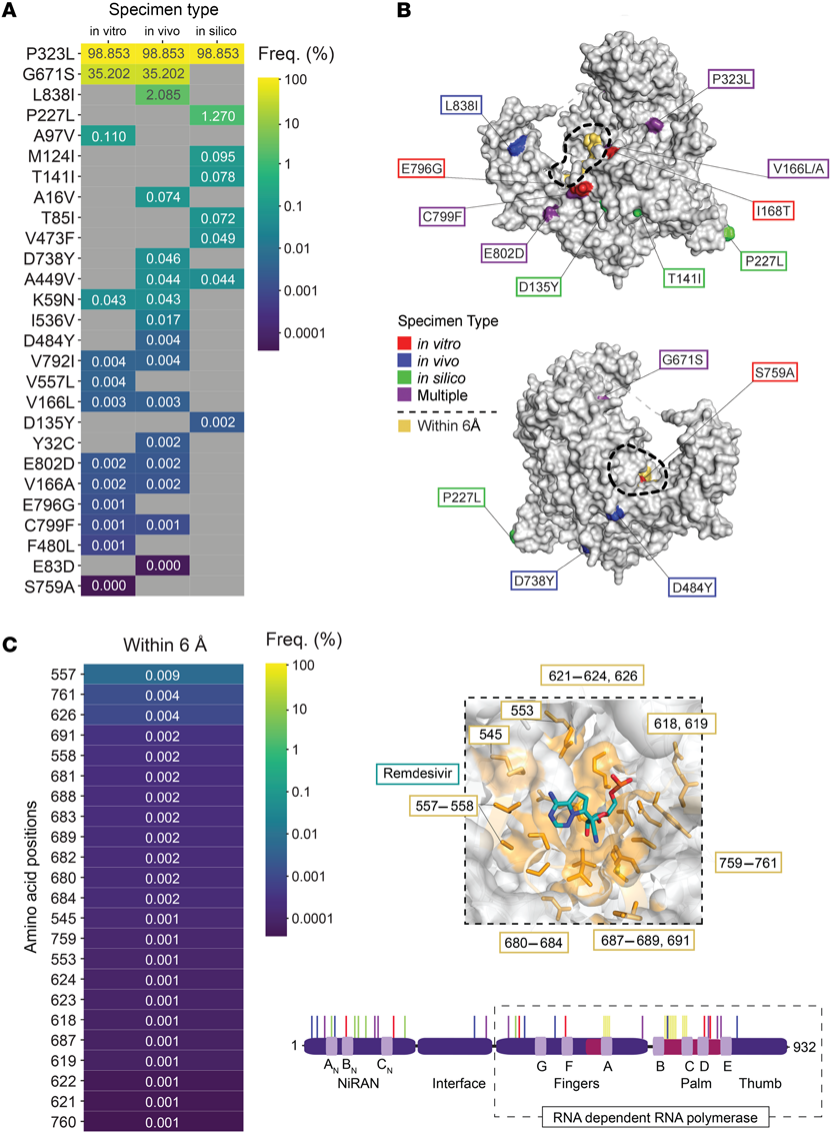
Frequency and position of nsp12 mutations previously implicated in remdesivir resistance. (**A**) Heatmap of nsp12 mutational frequency at positions previously implicated in remdesivir resistance through in vivo, in vitro, or in silico experimentation (reported as percentage of total SARS-CoV-2 sequences in the GISAID database, *n* = 15,998,330 sequences [accessed January 4, 2024]). (**B**) Structure of nsp12 (PDB 7BV2) highlighting the mutations reported in **A**. Residues within 6 Å of the remdesivir binding pocket are shaded in yellow and labeled in the magnified inset. A linear domain representation of nsp12 highlighting these mutations is shown below. (**C**) Heatmap of nsp12 mutational frequency (Freq.) at positions that interact with or are within 6 Å of remdesivir (reported as percentage of total SARS-CoV-2 sequences in the GISAID database, *n* = 15,998,330 sequences [accessed January 4, 2024]).

To assess the frequency with which these 29 putative resistance mutations have been observed in clinical isolates, we extracted all available SARS-CoV-2 sequences from the Global Initiative on Sharing All Influenza Data (GISAID) database (*n* = 15,998,330; accessed January 4, 2024) and calculated mutational frequency relative to the Wuhan-Hu-1 reference sequence (GenBank MN908947.3) (Figure 1A). Of the 29 unique mutations, only P323L was implicated by all 3 methodologies (in vitro, in vivo, and in silico), and it occurred at the highest frequency among all publicly available SARS-CoV-2 genome sequences (98.9%). This mutation emerged early in the COVID-19 pandemic alongside the Spike:D614G mutation and has been found in every subsequent SARS-CoV-2 lineage. Seven other mutations (K59N, V166A, V166L, G671S, V792I, C799F, and E802D) were found in both in vitro and in vivo studies. Most of these were extremely rare, occurring at a frequency of less than 0.1%, with the exception of G671S (35.2%), which was identified in several lineages of the Delta (B.1.617.2 and its descendants) and Omicron (BA.2.75 and its descendants) VOCs. The only other mutation to be implicated by at least 2 methodologies was A449V, which has only occurred at a low frequency (0.044%). The 20 other mutations identified were found by only a single methodology and occurred at a frequency less than 0.1%, with the exception of L838I (2.085%), P227L (1.27%), and A97V (0.11%).

Nsp12 consists of an N-terminal nidovirus RdRp-associated nucleotidyltransferase (NiRAN) domain, an RdRp domain, and an interface domain that links the two. The RdRp domain is conceptualized as a cupped right hand that contains finger, palm, and thumb domains with interspersed, conserved polymerase motifs (A–G) (49). he previously implicated remdesivir resistance mutations were found to be dispersed throughout the nsp12 structure (Protein Data Bank [PDB] 7BV2), with 13 mutations in the NiRAN domain, 14 mutations in the RdRp domain, and 2 mutations in the interface domain (Figure 1B) (49, 50). Notably, these mutations were all distant from the remdesivir binding site, with the exception of V557L and S759A (Figure 1B). In total, there are 23 residues in nsp12 that lie within 6 Å of the remdesivir binding site (51). While mutations of these residues could theoretically impact remdesivir efficacy, many of them are required for nucleotide and/or RNA binding and are highly conserved. The highest observed mutational frequency among these positions was 0.009% at residue V557 (Figure 1C), mutation of which had been previously link to remdesivir resistance (34). The only other residue implicated in remdesivir resistance (S759) had a low mutational frequency of only around 0.001%. No other proximal residue was implicated in a previous remdesivir resistance study or had a mutational frequency in the GISAID sequence database greater than 0.005%.

Taken together, these data suggest that mutations in and around the remdesivir binding site have too great a fitness cost relative to the selective pressure applied by remdesivir in vivo. On the contrary, a majority of previously implicated resistance mutations occur distal to the remdesivir binding site, suggesting a mode of action that may not directly affect remdesivir incorporation into viral RNA.

### SARS-CoV-2 exhibits similar genetic divergence in the presence and absence of remdesivir treatment.

**Figure 2:**
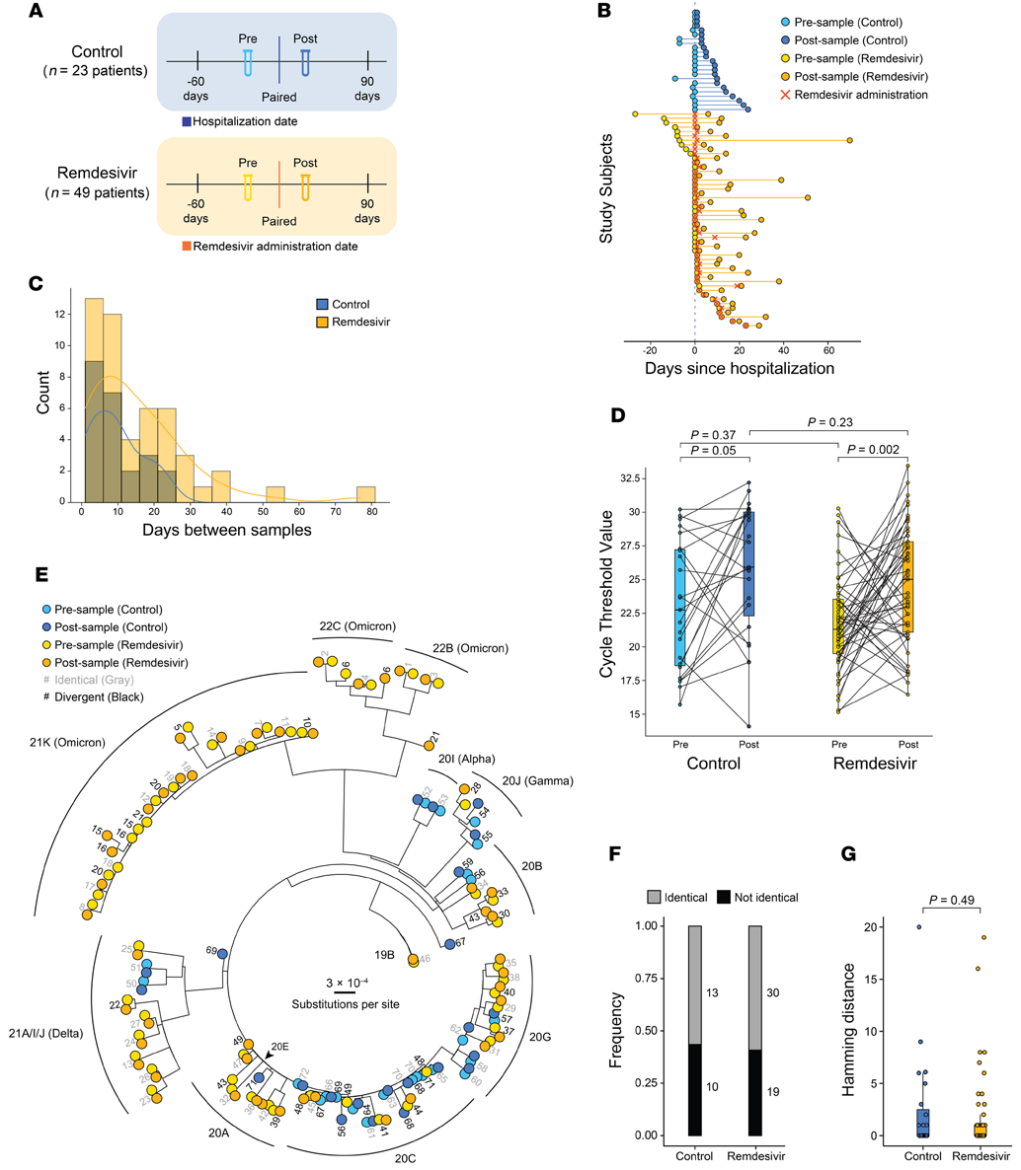
SARS-CoV-2 divergence in hospitalized patients with COVID-19 treated with and without remdesivir. (**A**) Schematic of the data filtering pipeline used to identify paired specimens for whole-genome sequencing that span hospitalization date in the control cohort (blue) or remdesivir administration date in the remdesivir cohort (orange). (**B**) Timeline of specimen collection for each patient in each cohort relative to hospitalization. The dotted line indicates the hospitalization date, and the “X” (dark orange) indicates remdesivir administration date. (**C**) Overlaid histogram and distribution curves of the days between the pre and post samples for each cohort. (**D**) SARS-CoV-2 N1Ct values for the paired pre and post specimens in each cohort as determined by qRT-PCR. Box-and-whisker plots represent the median (center line) and first/third quartiles (box), with tails extending 1.5 times the IQR. Specimens from the same patient are connected by lines. Statistical analysis for the paired samples within a cohort was conducted using Wilcoxon’s signed-rank test, and statistical analysis for the unpaired samples across cohorts was conducted using the Mann-Whitney *U* test, with *P* values indicated. (**E**) Phylogenetic tree of SARS-CoV-2 whole-genome sequences from each specimen in each cohort. Branch tips are colored by specimen type (pre versus post and cohort) and labeled by use of a randomized patient identifier. Gray patient labels denote identical consensus sequences in pre and post specimens, while black labels denote divergent sequences. Clade designations are specified on the outer ring. (**F**) Stacked bar chart showing the relative frequency of patients with identical (gray) versus divergent (black) pre and post sequences. The count is shown adjacent to each bar. (**G**) Hamming distance between paired pre and post specimens in each cohort depicted as a box-and-whisker plot representing the median (center line) and first/third quartiles (box), with tails extending 1.5 times the IQR. Statistical analysis was conducted using a linear model with a negative binomial distribution controlling for time between specimens.

A majority of studies looking for remdesivir resistance mutations in vivo were limited to single case studies or small cohorts and lacked a control cohort of individuals who did not receive remdesivir. To better assess the frequency of previously identified remdesivir resistance mutations among hospitalized patients with COVID-19 who did or did not receive remdesivir, we performed a single-center retrospective cohort study of inpatients who tested positive for SARS-CoV-2 at a Northwestern Medicine–affiliated (NM-affiliated) hospital between March 1, 2020, and March 1, 2023. Electronic health record data collected through NM’s centralized repository was used to identify patients who had been hospitalized in the NM system for whom there was at least 1 banked SARS-CoV-2–positive specimen collected within 60 days prior to remdesivir administration (termed “pre”) and at least 1 banked specimen collected within 90 days after remdesivir administration (termed “post”). The same parameters were used to identify a control cohort of hospitalized patients who did not receive remdesivir using hospital admission date as a stand-in for remdesivir administration date, with the rationale that most patients who received remdesivir did so on or near the date of admission (Figure 2, A and B).

Specimens with sufficient SARS-CoV-2 viral load (Ct value less than 33 as determined by quantitative reverse transcription PCR [qRT-PCR]) were subjected to whole-genome sequencing using a multiplex PCR amplicon strategy per the ARTIC protocol (52). Nextclade and PANGOLIN tools were used to assign clade and lineage designations, respectively (53, 54) (clade/lineage designations and accession numbers available in Supplemental Table 2). Only patients with at least 1 pre and 1 post sequence were considered in this analysis. This resulted in a remdesivir cohort of 49 patients with paired sequences before and after remdesivir administration; and a control cohort of 23 patients not treated with remdesivir with paired sequences before and after hospital admission (Figure 2B). The distribution of days between sampling for both cohorts roughly followed a log-normal distribution, though the remdesivir cohort skewed to slightly longer times between the pre and post specimens (Figure 2C). The remdesivir cohort also skewed slightly older (median age 68 years [IQR 56, 75] compared with 63 [IQR 42, 70]), was predominantly White (59.2% compared with 30.5%), and had more female patients (40.8% compared with 26.1%) (Table 1). The cohort that received remdesivir also had notably more comorbidities (median of 4 compared with 1). Some of these differences reflect known risk factors for severe COVID-19 that may have influenced the clinical decision to administer remdesivir. Regardless, the two cohorts had comparable clinical outcomes, with 76% and 83% of patients in the remdesivir and control cohorts requiring intensive care, respectively. Finally, we note that the remdesivir cohort had a higher proportion of Delta and Omicron infections compared with the control cohort (Table 2), likely representing the fact that remdesivir became the standard of care for inpatients by the time those two VOCs emerged.

In both cohorts, the pre sample had a significantly lower Ct value (higher viral load) compared with the post sample, consistent with previous studies (Figure 2D) (55–57). The Ct values of the pre and the post specimens across cohorts were not significantly different. Next, we used a maximum-likelihood (ML) phylogenetic analysis to assess genetic divergence between paired sequences in both cohorts (Figure 2E). Of the 72 total patients, 43 had no divergence in consensus viral sequence between the pre and post specimen (13 in the control cohort and 30 in the remdesivir cohort) (Figure 2E, gray identifiers). The other 29 patients had at least 1 mutation arise between their pre and post samples (Figure 2E, black; mutations listed in Supplemental Table 3). Only 6 patients had a mutation arise in nsp12, including some mutations that had been previously implicated in remdesivir resistance (G671S, P323L). Notably, however, these arose in both the control and remdesivir cohorts. Overall, the frequency of patients with no divergence over time was markedly similar (Figure 2F). To further quantify this, we calculated the hamming distance, a metric of genetic divergence, by counting the mutations between paired sequences. We then used a linear mixed-effects model to test for a statistical difference between the cohorts while controlling for time between sampling (Figure 2G). We observed no significant difference between cohorts, suggesting that remdesivir treatment was not driving viral genetic divergence in patients at the consensus genome level.

### SARS-CoV-2 intra-host genetic diversity increases following remdesivir treatment in specific ORFs.

**Figure 3:**
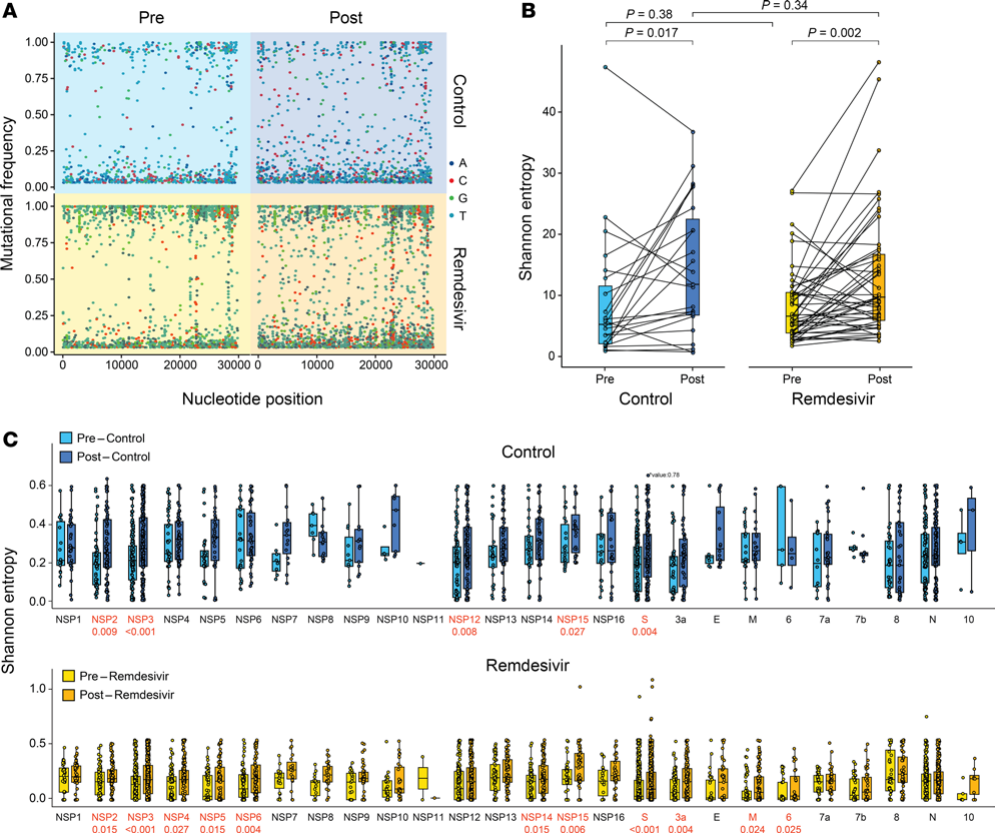
Changes in SARS-CoV-2 genetic diversity after remdesivir administration. (**A**) Dot plots depicting mutational frequency per position across the SARS-CoV-2 genome relative to the Wuhan-Hu-1 reference genome, calculated as the fraction of raw reads and colored by nucleotide. All specimens within a group are overlaid in a single plot. Mutational frequencies less than 1% are not shown. (**B**) Shannon entropy [*Sh* = *SUM*[–(*pi*) * log_2_(*pi*)], where *pi* where is nucleotide frequency] across the genome of each isolate; paired specimens are connected by lines. Statistical analysis was conducted using a linear mixed-effects model controlling for time between paired specimens and within-patient variability. (**C**) Shannon entropy per nucleotide position within each viral ORF. Statistical analysis was conducted using a linear mixed-effects model while controlling for time between samples, gene, and within-patient variability for each patient. For **B** and **C**, box-and-whisker plots represent the median (center line) and first/third quartiles (box), with tails extending 1.5 times the IQR. *P* values were adjusted for FDR using the Benjamini-Hochberg method; differences at positions with an adjusted *P* value less than 0.05 were considered significant (values shown in red). M, membrane.

Next, we aimed to characterize intra-host genetic diversity, utilizing individual reads to calculate mutational frequency by position and help capture mutations that were not evident at the consensus genome level. First, we calculated the mutational frequency at each position relative to the SARS-CoV-2 reference genome (Wuhan-Hu-1) in each patient isolate, where 0 indicates that all reads match the reference and 1 indicates that all reads differ from the reference. Mutational frequency was plotted by genomic position, and the resultant dot plots from each specimen were overlaid with the others from their respective category (Figure 3A). In both cohorts, we observed more diversity in the post specimens (i.e., more positions with higher levels of mutational frequency), most notably around the Spike ORF (positions 21,563–25,384). To determine whether these differences were statistically significant, we used mutational frequency to calculate the Shannon entropy value of each isolate (Figure 3B). Shannon entropy is a measurement of the positional diversity independent of a reference; in the present study, if 100% of reads corresponded to 1 nucleotide, the entropy was defined as 0, and if it is an even split between all 4 nucleotides, the entropy was 1. As this measurement is independent of a reference sequence, it is clade agnostic. A linear mixed-effects model was used to compare Shannon entropy in the pre and post specimens in each cohort while controlling for time between sampling and within-patient variability of paired samples. This model showed a significant increase in genetic diversity in the post compared with the pre specimens within each cohort, but found no statistical difference in Shannon entropy when comparing pre or post specimens between cohorts (Figure 3B).

These data suggest that the overall degree by which SARS-CoV-2 diversifies over time in a host is not affected by remdesivir administration, but they do not rule out potential differences in where this diversification is taking place. To better assess this, we applied the same statistical framework to look for differences in Shannon entropy between the pre and post samples within each SARS-CoV-2 ORF and nsp coding region (Figure 3C). These data indicated that increased Shannon entropy over time was concentrated in specific ORFs and nsps. In the control cohort, diversity was significantly increased over time in nsp2, nsp3, nsp12, nsp15, and Spike (gene names and *P* values in red). These same regions saw increased diversity in the remdesivir cohort (though the difference in nsp12 was borderline insignificant; *P* = 0.07). However, in the remdesivir cohort, diversity was also significantly increased in nsp4, nsp5, nsp6, nsp14, ORF3a, membrane, and ORF6. Taken together, these data suggest that while the overall degree of genetic diversification over time was not affected by remdesivir administration, remdesivir may result in the preferential accumulation of variation in specific ORFs.

### Genetic diversity increases at specific nucleotide positions after remdesivir treatment.

**Figure 4:**
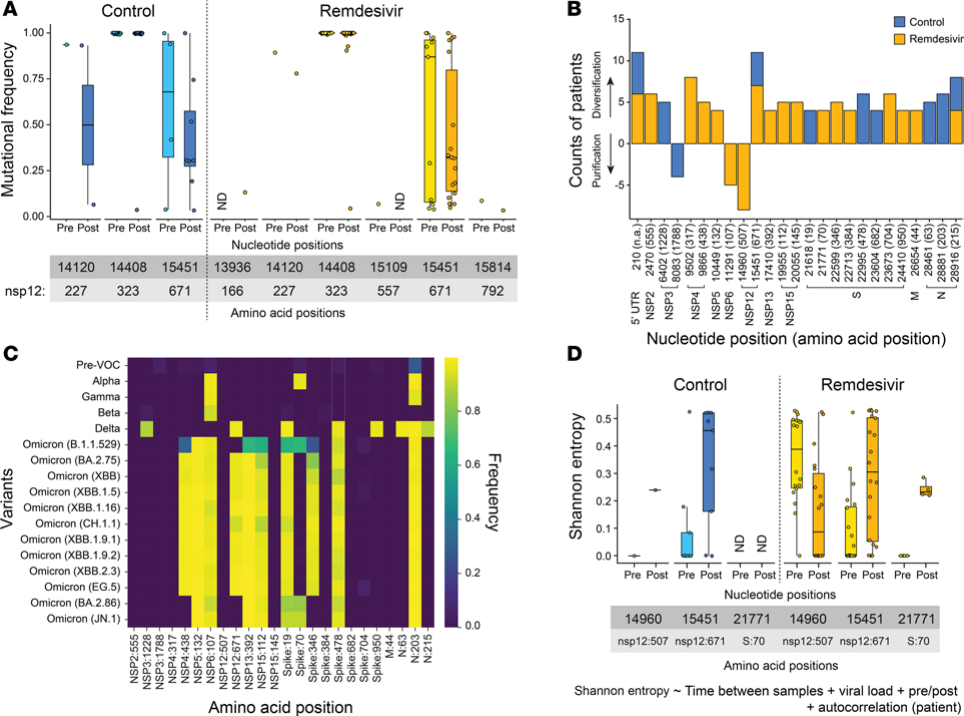
Identification of nucleotide positions with significantly altered genetic diversity after remdesivir administration. (**A**) Mutational frequency at positions in nsp12 previously implicated in remdesivir resistance relative to the Wuhan-Hu-1 reference genome in pre and post specimens from each cohort. Nucleotide and amino acid positions are shown below. Box-and-whisker plots represent the median (center line) and first/third quartiles (box), with tails extending 1.5 times the IQR. Only specimens with greater than 1% mutational frequency at a given position are shown (positions that were not detected are indicated as “ND”). (**B**) Stacked bar plot illustrating the number of patients in each cohort that exhibited a change in Shannon entropy at a given position in paired specimens. To be shown, at least 4 patients in either cohort had to have an increase in Shannon entropy from 0 (i.e., diversification) or decrease to 0 (i.e., purification). (**C**) Heatmap of mutational frequency at positions identified in **B** by lineage (reported as percentage of total SARS-CoV-2 sequences per variant in the GISAID database [accessed January 4, 2024]). (**D**) Shannon entropy in pre and post specimens from each cohort at 3 nucleotide positions that had significant changes following remdesivir administration. Box-and-whisker plots represent the median (center line) and first/third quartiles (box), with tails extending 1.5 times the IQR. Significantly changing positions in the remdesivir cohort were identified using a linear mixed-effects model (specified below the graph) controlling for time between paired specimens, viral load, and within-patient variability for each patient. *P* values were adjusted for FDR using the Benjamini-Hochberg method; differences at positions with an adjusted *P* value less than 0.1 were considered significant.

To determine whether remdesivir changes the degree of SARS-CoV-2 diversification at specific residues, we first examined mutational frequency at each nucleotide position corresponding to an amino acid either previously implicated in remdesivir resistance or within 6 Å of the remdesivir binding site (Figure 1). Mutational frequencies less than 1% are not shown; nucleotide positions that did not reach at least 1% mutational frequency in at least 1 specimen are likewise not shown. Notably, very little diversity was observed at any of the previously identified positions. In the control cohort, some mutations were seen at positions 14,120 (nsp12:227) and 15,451 (nsp12:671) in both pre and post specimens (Figure 4A). Position 14,408 (nsp12:323) was mutated in nearly every isolate, reflective of the nsp12:P323L mutation that became fixed in the SARS-CoV-2 population early in the pandemic (58, 59). In the remdesivir cohort, these same mutations were observed, though many more isolates showed diversity at 15,451 (nsp12:671) in the post specimens (10 pre isolates and 18 post isolates). Note that 5 specimens in the pre and post groups had high mutational frequency at this position due to a preexisting nsp12:G671S mutation in the Delta VOC. Low-level diversity was also seen at positions 13,936 (nsp12:166), 15,109 (nsp12:557), and 15,814 (nsp12:792). Notably, no mutations in either cohort were observed at 21 of the 27 previously identified resistance sites and at 22 of the 23 sites within 6 Å of the remdesivir binding pocket. Overall, these results confirm the low incidence of mutations previously implicated in remdesivir resistance, even in minority populations of the virus within a host.

We next used an unbiased approach to screen for potential sites of intra-host diversification across the genome. Specifically, we looked for positions in paired specimens where Shannon entropy increased from 0 (i.e., diversification) or decreased to 0 (i.e., purification) in at least 4 patients (Figure 4B). In total, we identified 25 positions across the genome that met these criteria, 22 of which showed evidence of diversification, consistent with our prior measures of increased diversity over time. Mutational frequency and Shannon entropy at each position in each cohort are shown in Supplemental Figure 1. Most of these positions were in the previously identified ORFs and nsps that showed evidence of diversification (Figure 3C). Three positions were found to diversify in both cohorts, including positions 210 (in the 5′-UTR), 15,451 (nsp12:671), 28,916 (nucleocapsid:215 [N:215]). Seven positions were specific to the control cohort (6 diversifying and 1 purifying), while 15 were specific to the remdesivir cohort (13 diversifying and 2 purifying). Only 2 positions were identified in nsp12, namely, 15,451 (nsp12:671), which showed evidence of diversification in both cohorts, and 14,960 (nsp12:507), which showed evidence of purification in the remdesivir cohort specifically. Each position undergoing selection was identified in at least 2 or more variant backgrounds, suggesting that these are not specific to one lineage (Supplemental Figure 2A).

Several of these positions were associated with specific mutations in different VOCs that have been shown to confer enhanced viral fitness (i.e., N:203, nsp12:671, etc.) (60–62). To begin to discern the evolutionary history at these positions, we calculated the frequency of nonsynonymous mutations at the corresponding amino acid positions in different VOCs (based on the GISAID database, accessed January 4, 2024) (Figure 4C). Remarkably, 15 of the 25 identified positions were associated with amino acid variations in 1 or more VOCs. Five residues were specifically associated with Omicron lineages (nsp4:438, nsp5:132, nsp13:392, nsp15:112, and Spike:346), 4 were associated with Delta lineages (nsp3:1228, Spike:950, N:63, and N:215), and 3 were found in both (nsp12:671, Spike:19, and Spike:478). Three residues were associated with earlier VOCs, including nsp6:107, Spike:70, and N:203. Nine positions (nsp2:555, nsp3:1788, nsp4:317, nsp12:507, nsp15:145, Spike:384, Spike:682, Spike:704, M:44), including 2 of the 3 sites that appeared to decrease diversity, were either never observed or were observed at an extremely low frequency across all variants. Intra-host divergence at these positions and their later emergence in VOCs over the course of the pandemic may be suggestive of enhanced fitness.

To assess whether increased diversity after remdesivir administration was statistically significant at any of these positions in our cohort, we employed a linear mixed-effects model to assess variations in Shannon entropy at each position in the remdesivir cohort while controlling for time between samples, viral load, and within-patient variability. Subsequently, we applied a correction for FDR to all *P* values using the Benjamini-Hochberg method and considered adjusted *P* values less than 0.1 as significant due to the high number of comparisons performed. This analysis identified 3 positions with statistically significant changes in diversity after remdesivir administration: 14,960 (nsp12:507), 15,451 (nsp12:671), and 21,771 (Spike:70) (Figure 4D). In our remdesivir cohort, positions 15,451 (nsp12:671) and 21,771 (Spike:70) exhibited an increase in Shannon entropy in the post specimen (increased diversity), while position 14,960 (nsp12:507) showed a decrease in Shannon entropy (decreased diversity). These changes are reflected in the nucleotide frequency at these positions in each patient (Supplemental Figure 2B). Taken together, these data suggest that remdesivir administration preferentially selects for SARS-CoV-2 diversification at several residues across the genome, many of which have been associated with VOCs and linked with enhanced viral fitness in other studies.

### Validation cohort containing only post samples confirms previous findings.

**Figure 5:**
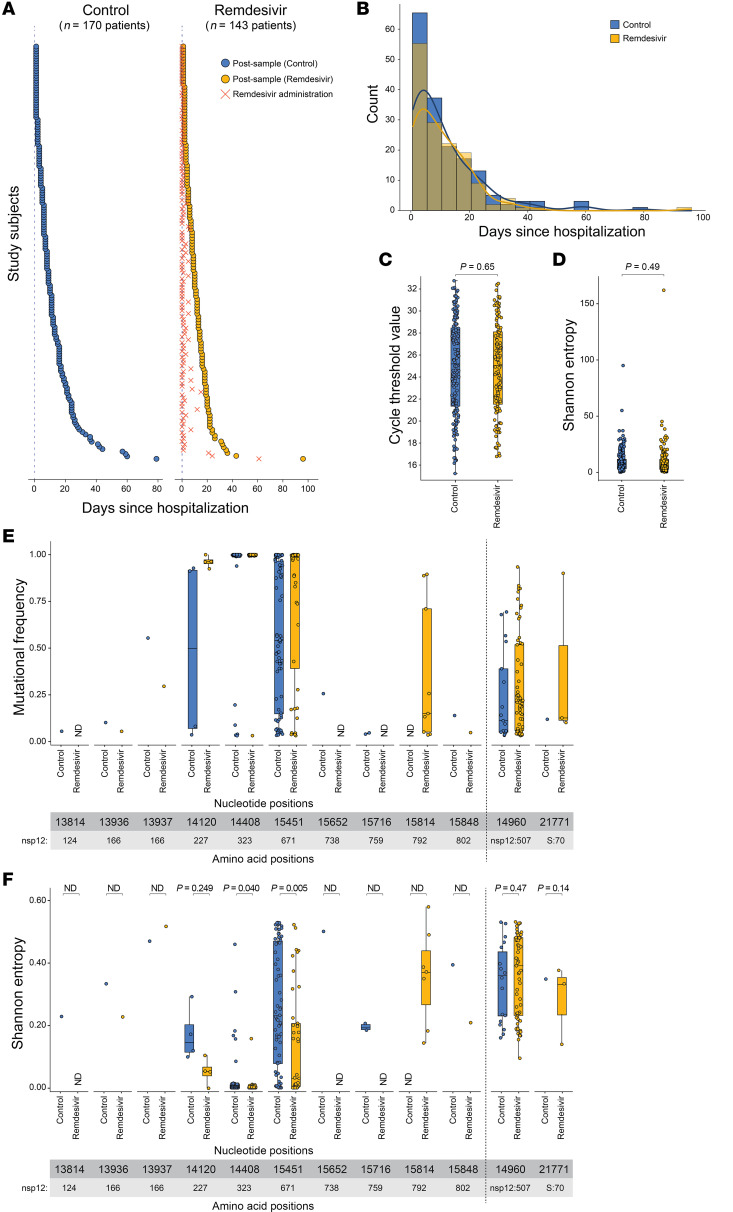
Comparison of nsp12 mutational frequency in patients who received remdesivir compared with a control cohort. (**A**) Timeline of specimen collection for each patient in each cohort relative to hospitalization. The dotted line indicates the hospitalization date, and the “X” (dark orange) indicates remdesivir administration date. (**B**) Overlaid histogram and distribution curves of the days between sample collection and hospitalization date for each cohort. (**C**) SARS-CoV-2 N1 Ct values for post specimens in each cohort as determined by qRT-PCR. Box-and-whisker plots represent the median (center line) and first/third quartiles (box), with tails extending 1.5 times the IQR. Statistical analysis was conducted using the Mann-Whitney *U* test, with *P* values indicated. (**D**) Shannon entropy across the genome of each isolate. Statistical analysis was conducted using a linear model while controlling for time between samples, viral load, and clade. (**E**) Mutational frequency at positions in nsp12 previously implicated in remdesivir resistance relative to the Wuhan-Hu-1 reference genome in the post specimen–only cohorts. Two additional positions identified from the analyses in Figure 4 are visualized on the right. Box-and-whisker plots represent the median (center line) and first/third quartiles (box), with tails extending 1.5 times the IQR. Only specimens with greater than 1% mutational frequency at a given position are shown (positions that were not detected are indicated as “ND”). (**F**) Shannon entropy at the same nucleotide positions as in **E** in the post specimen–only cohorts. Box-and-whisker plots represent the median (center line) and first/third quartiles (box), with tails extending 1.5 times the IQR. Statistical analysis was conducted using a linear model while controlling for time between samples and viral load (except for position 21771, which had too few data points to fit a model that also included viral load).

To validate these findings, we returned to the electronic health record data to identify an independent cohort of inpatients for which we had at least 1 post specimen collected within 90 days after remdesivir administration. We likewise identified an independent control cohort for which we had at least 1 post specimen collected within 90 days after hospitalization (Figure 5A). Specimens were subject to viral whole-genome sequencing as above (accession numbers provided in Supplemental Table 2); this resulted in a remdesivir cohort of 143 patients and a control cohort of 170 patients, each represented by a single, unpaired post specimen. The distribution of days between hospitalization and sample collection was highly comparable between cohorts (Figure 5B). Again, the remdesivir cohort skewed slightly older (median age 63 compared with 57) and had more comorbidities (median of 4 compared with 2), though with more comparable distributions by sex and race (Table 1). The cohorts had comparable clinical outcomes, with 69% and 65% of patients in the remdesivir and control cohorts requiring intensive care, respectively. There was no significant difference in viral load (N1 Ct value) between specimens in either cohort (Figure 5C). As before, there was a greater proportion of Omicron specimens in the remdesivir cohort (Table 2), likely reflecting the change in standard of care for hospitalized COVID-19 patients between pandemic waves. Note that while our last analyses accounted for clade by always comparing mutations between a pre and post specimen from the same patient, here this was not an option and so we account for clade explicitly in our statistical modeling.

We first calculated genome-wide Shannon entropy as a measure of intra-host diversity in each specimen. No significant difference was observed between cohorts when using a linear mixed-effects model controlling for time since hospitalization, viral load, and clade (Figure 5D), similar to what was observed in the last cohort. We next examined mutational frequency at each nucleotide position corresponding to an amino acid either previously implicated in remdesivir resistance or within 6 Å of the remdesivir binding site (Figure 1). Only 10 positions showed a mutational frequency greater than 1% in at least 1 specimen (Figure 5E). Position 14,408 (nsp12:323) was mutated in nearly every isolate in both cohorts, again reflective of the nsp12:P323L mutation. Two positions at 14,120 (nsp12:227) and 15,451 (nsp12:671) had higher mutational frequencies in the remdesivir compared with the control cohort, and both were also observed in the prior paired cohort (though position 14,120 was observed equally frequently in the pre and post specimens). Notably, mutations at position 15,814 (nsp12:792) were found only in the remdesivir cohort (*n* = 9 specimens), suggestive of potential selection at this site. The other mutations were either found in only the control cohort (at positions 13,814 [nsp12:124], 15,652 [nsp12:738], and 15,716 [nsp12:759]) or were found only once in each cohort (at positions 13,936 [nsp12:166], 13,937 [nsp12:166], and 15,848 [nsp12:802]). Notably, mutational frequency increased at each of the positions that were found to significantly change in our paired cohort (14,960 [nsp12:570], 15,451 [nsp12:671], and 21,771 [S:70]), though mutations at position 21,771 (S:70) were relatively rare.

Finally, we calculated the Shannon entropy at each of these positions and used a linear mixed effects model to assess for statistical significance between cohorts while controlling for time since hospitalization, viral load, and clade (Figure 5F). The data were too sparse to calculate significance at positions 13,814, 13,936, 13,937, 15,652, 15,716, and 15,848. There was no statistically significant difference in Shannon entropy at positions 14,120 (nsp12:227), 14,960 (nsp12:570), and 21,771 (S:70). Shannon entropy at position 15,814 (nsp12:792) could not be statistically compared between cohorts, as it only appeared in the remdesivir cohort. However, position 15,451 (nsp12:671) had significantly lower entropy in the remdesivir compared with the control cohort, reflective of there being more specimens with near 100% mutational frequency. Taken together, these data suggest that while most previously identified remdesivir resistance mutations are either rarely occurring or have not been specifically associated with remdesivir administration, variants at 2 positions (15,451 [nsp12:671] and 15,814 [nsp12:792]) may be selected for by remdesivir in vivo.

## Discussion

In this single-center, retrospective cohort study, we assessed SARS-CoV-2 divergence and intra-host diversity over time in a cohort of hospitalized inpatients who received remdesivir (*n* = 192) alongside a control cohort of hospitalized inpatients who did not (*n* = 193). These data revealed that most mutations that had been previously associated with remdesivir resistance in nsp12 were exceedingly rare and were equally likely to emerge in patients who did and did not receive remdesivir. The exception to this was increased diversity at positions 15,451 (nsp12:671) and 15,814 (nsp12:792). Independent analysis of viral diversification before and after remdesivir administration revealed several additional positions under purifying or diversifying selection, many of which arose in 1 or more VOCs. Diversity at two of these positions (14,960 [nsp12:570] and 21,771 [S:70]) was statistically significantly different and reflect what we believe to be novel mutations associated with remdesivir treatment. These 4 mutations are not anticipated to directly impact remdesivir incorporation but may confer enhanced replicative fitness as an indirect means to overcome this selective pressure.

Prior studies have attempted to identify remdesivir-induced resistance in treated patients, but most have done so through case studies in immunocompromised individuals with COVID-19. For example, Gandhi et al. detailed the emergence of an nsp12:E802D mutation in a patient with persistent infection following remdesivir administration. This mutation conferred a 6-fold increase in remdesivir resistance when measured in vitro (29). However, the case patient, a 70-year-old woman with stage IV non-Hodgkin’s lymphoma, also had both lymphocytopenia and hypogammaglobulinemia, which obfuscated the factors driving the emergence of this mutation and the likelihood of its appearance. Furthermore, the study concluded that the nsp12:E802D mutation came at a fitness cost to the virus when not in the presence of remdesivir, suggesting that this mutation is unlikely to propagate broadly. This result was echoed by Nirmalarajah et al., who collected 109 samples from 44 patients (14 patients received remdesivir and 30 patients did not) and analyzed sequences for mutational changes at the consensus genome level as well as at the minority level intra-host (63). They detected only stochastic, low-frequency variants that were not likely driven by remdesivir and were unlikely to spread broadly.

In this study, we sought to expand this approach to a much larger cohort of patients who did and did not receive remdesivir. Our findings largely echoed those of these prior studies, in that a majority of previously identified remdesivir resistance mutations were never observed or were observed as frequently in the control as in the remdesivir cohorts. Likewise, very few mutations in and around the remdesivir binding site were observed, likely reflecting the high fitness cost of these mutations in vivo. Consistent with this, overall levels of viral divergence and intra-host viral diversity were unchanged by remdesivir administration. However, we did find increased mutational frequency in specific ORFs and at specific positions in our remdesivir cohorts. From our first cohort of patients, for whom we had paired specimens before and after remdesivir treatment, we identified 3 positions that had significantly different intra-host diversity following remdesivir treatment: 14,960 (nsp12:507), 15,451 (nsp12:671), and 21,771 (Spike:70).

Position 15,451 (nsp12:671) has been extensively studied, as the G15451A (nsp12:G671S) mutation is a defining mutation for many Delta and Omicron lineages. Pitts et al. explicitly tested the potency of remdesivir against SARS-CoV-2 viruses harboring either the nsp12:P323L mutation or the nsp12:P323L/G671S double mutation and found no significant difference in the drug’s EC_50_ (35). Kim et al. explored this further, finding that the P323L/G671S double mutation enhances nsp12-nsp8 binding and increases overall replicative fitness of the virus in vivo (60). Position 21,771 (S:70) has likewise been widely studied, as deletion of this amino acid was a defining mutation in the Alpha VOC (64). Deletion of residues 69 and 70 (ΔH69/V70) was found to enhance incorporation of the Spike protein into viral particles and enhance viral infectivity (64). Position 14,960 (nsp12:507) has not been as widely studied and has not been previously associated with any VOC. Unlike the other 2 positions, which underwent diversification, this position had significantly decreased Shannon entropy following remdesivir administration, suggestive of purifying selection. This position has been implicated in stabilizing the phosphate backbone of the RNA template, mutation of which decreases RdRp activity (65, 66). In all 3 of these cases, we saw selective pressure at sites that confer enhanced viral fitness. This suggests that while mutations at the remdesivir binding site may come at too high a fitness cost to emerge in vivo, the drug may enhance the selective pressure for mutations that confer greater overall fitness as an indirect way to overcome the drug.

In our second cohort of patients for which we lacked paired specimens, we found a fourth position that had enhanced intra-host diversity after remdesivir administration. Position 15,814 (nsp12:792) exhibited an increase in mutational frequency in 9 isolates from our remdesivir cohort and was never mutated in any control specimen. In vitro selection experiments by Stevens et al. previously identified nsp12:V792I as a remdesivir resistance mutation that works by improving the incorporation of UTP opposite the incorporated remdesivir monophosphate (31). In vitro, nsp12:V792I was often selected alongside a second site mutation, nsp12:S759A, which decreased RdRp selectivity for remdesivir as a substrate. These 2 mutations were never observed together in any of our isolates, and nsp12:S759A was only observed twice, both in control specimens. However, given the fact that nsp12:V792I can directly act to diminish remdesivir efficacy and does not come at too high a fitness cost to arise in vivo, it should be carefully monitored. If the nsp12:V792I mutation were to arise in a contemporary variant with enhanced transmissibility or immune evasion, it could result in widespread antiviral resistance.

Taken together, these results suggest that the high fitness cost of mutations in and around the remdesivir binding site limit their emergence in vivo. In contrast, remdesivir exerts a selective pressure for enhanced replicative fitness that may enable intra-host persistence despite antiviral therapy. Indeed, while mutations in and around the remdesivir binding site have never emerged in any circulating lineages, several positions in which we observe increased intra-host diversity after remdesivir treatment have been mutated in different VOC lineages. Notably, this includes several mutations outside of Spike and nsp12. This suggests that continued surveillance of minority variants that emerge in hospitalized inpatients could enable detection of mutations that provide enhanced viral fitness. 9 of these positions have yet to emerge as a lineage-defining mutation and their prevalence in new emerging lineages should be carefully monitored.

This study has several limitations. First, samples were collected at a single center and may not be representative of the effect of remdesivir on SARS-CoV-2 variation in the broader population. While this is the largest cohort of COVID-19 patients used to study remdesivir-driven mutations, the demographics are skewed toward the population served by Northwestern Memorial Hospital in Chicago. Second, we were not powered to separate out these results by patient immune status. It remains unclear whether the mutations that arise in immunocompetent individuals are comparable to those in immunosuppressed or immunocompromised individuals, who often have higher mutational burdens due to higher viral loads and more persistent infection (67–69). Third, while we controlled for the time between sampling and/or the time between sample collection and hospitalization, these may not be directly comparable between cohorts. Our use of hospitalization date as a stand-in for remdesivir administration date for our control cohort relied on the fact that most patients who received remdesivir did so shortly after admission, though this situation was far from universal. Finally, baseline differences in the viral sequence over time were a major challenge in the analysis, especially as remdesivir was more likely to be administered to patients infected with later variants (i.e., the Delta or Omicron VOCs). We controlled for this by comparing paired pre and post samples within a patient, by using Shannon entropy as a reference-agnostic measure of diversity, and by controlling for clade in our models where possible. However, we cannot rule out strain-specific effects in all cases.

Overall, this study describes the effect of remdesivir on SARS-CoV-2 diversity and evolution in what we believe to be the largest patient cohort to date. Our results suggest that SARS-CoV-2 variants with enhanced replicative fitness may be selected for in the presence of antiviral therapy as an indirect means to overcome this selective pressure. While most resistance mutations are exceedingly rare in vivo, the nsp12:V792I mutation in particular warrants continued monitoring. This study underscores the multifaceted channels through which a virus may mutate to ensure its survival and transmission. Continued surveillance efforts in patient populations will be vital to identifying emerging variants with fitness benefits and/or altered sensitivities to therapeutics.

## Methods

### Sex as a biological variable.

Our study examined both male and female patients, and similar findings are reported for both sexes. Refer to Tables 1 and 2 for patient demographics.

### Ethics statement.

All handling and extraction of patient data was approved through the Northwestern University Internal Review Board (IRB ID: STU00206850, STU00212260, STU00212267). Personal health information (PHI) was removed prior to analysis and samples were coded with a unique identifier. Unique identifiers were maintained by select study personnel approved by the IRB and were stored on a secure, protected server. Data including PHI are stored on a secure server in compliance with HIPAA requirements, with accessibility limited to study team members approved by the IRB.

### Clinical data extraction.

Early in the COVID-19 pandemic, IRB approval (STU00212267) was obtained to create a data mart of all adults diagnosed and treated for COVID-19 across NM using the NM Enterprise Data Warehouse (NMEDW). The NMEDW is a joint initiative across Northwestern University Feinberg School of Medicine and Northwestern Memorial Healthcare Corporation to create a single, comprehensive, and integrated repository of all clinical and research data sources to facilitate research, clinical quality, and healthcare operations. The following electronic data elements were extracted and compiled once a week from the NMEDW for all adults diagnosed with COVID-19: demographics, health system visits/level of care (i.e., outpatient, emergency department, hospital, ICU), vital signs, specific laboratory test results, imaging studies, comorbid conditions/diagnoses (via International Classification of Diseases, Ninth Edition [ICD-9]/ICD-10 coding), pharmacologic therapy (initially via NMEDW pharmacy/medication records, then confirmed as needed with electronic chart review).

### Sample collection and viral load determination.

Residual diagnostic specimens from individuals testing positive for SARS-CoV-2 in the NM healthcare system were collected as part of an established biobank in the Center for Pathogen Genomics and Microbial Evolution at the Northwestern University Feinberg School of Medicine. Samples collected between March 1, 2020, and March 1, 2023, were included as part of this study (protocols STU00206850 and STU00212260). Electronic health record data collected through NM’s centralized repository were used to identify patients who had been hospitalized at Northwestern Memorial Hospital in Chicago for whom we had at least 1 banked SARS-CoV-2–positive respiratory specimen collected within 60 days prior to remdesivir administration and at least 1 banked specimen collected within 90 days after remdesivir administration. For patients who received multiple doses of remdesivir, only specimens flanking the first dose were considered for these analyses. The same parameters were used to identify a control cohort of hospitalized patients who did not receive remdesivir, with hospital admission date using as a stand-in for remdesivir administration date.

Viral RNA was extracted from nasopharyngeal specimens stored in viral transport medium (VTM) utilizing the QIAamp 96 Virus QIAcube HT Kit (QIAGEN); VTM-only controls were included in each extraction. Laboratory testing for the presence of SARS-CoV-2 was performed by qRT-PCR with the CDC 2019-nCoV RT-PCR Diagnostic Panel utilizing N1 and RNase P probes as previously described (70). Positive and negative controls for SARS-CoV-2 and RNase P were included in each qRT-PCR experiment alongside the VTM-only sample from the RNA extraction, a no-template control, and standard curves for SARS-CoV-2 and RNase P. Specimens with RNase P Ct value above 35 were of insufficient quality and were excluded from future studies. N1 Ct values less than or equal to 35 were considered positive, these Ct values were used in all subsequent analyses.

### cDNA synthesis and viral genome amplification.

cDNA synthesis was performed with SuperScript IV First Strand Synthesis Kit (Thermo) using random hexamer primers according to manufacturer’s specifications. Direct amplification of the viral genome cDNA was performed in multiplexed PCR reactions to generate approximately 400 bp amplicons tiled across the genome. The multiplex primer set, comprising 2 non-overlapping primer pools, was created using PrimalScheme and provided by the ARTIC Network (versions 4, and 4.1 releases) (https://www.protocols.io/view/ncov-2019-sequencing-protocol-bp2l6n26rgqe/v1). PCR amplification was carried out using Q5 Hot Start HF Taq Polymerase (NEB) with 5 μL cDNA in a 25 μL reaction volume. A 2-step PCR program was used, with an initial step of 98°C for 30 seconds, then 35 cycles of 98°C for 15 seconds, followed by 5 minutes at 65°C. Separate reactions were carried out for each primer pool and validated by agarose gel electrophoresis.

### Sequencing library preparation and whole-genome sequencing.

This study utilized a seqWell plexWell 384 kit, per the manufacturer’s directions, for library preparation prior to sequencing on the Illumina MiSeq platform. Pooled libraries were sequenced on the Illumina MiSeq using the V2 500 cycle kit.

Viral genome consensus sequences were determined from sequencing reads as previously described. Sequencing reads were trimmed to remove adapters and low-quality sequences using Trimmomatic v0.36. Trimmed reads were aligned to the reference genome sequence of SARS-CoV-2 (MN908947.3) using Burrows-Wheeler Aligner (BWA) v0.7.15. Pileups were generated from the alignment using SAMtools v1.9, and consensus sequence was determined using iVar v1.2.2, with a minimum depth of 10, a minimum base quality score of 20, a consensus frequency threshold of 0 (i.e., majority base as the consensus), and a maximum number of 3,000 uncalled positions. To validate our sequencing results and rule out potential contamination, we randomly selected 5% of all samples for repeat sequencing, including an independent RNA extraction, cDNA synthesis, multiplex PCR amplification, library preparation, and Illumina sequencing run. All repeat sequencing results for this study resulted in identical lineage designations.

### Phylogenetic analysis.

Nextclade and PANGOLIN tools were used to assign clade and lineage designations, respectively (53, 54). Consensus viral genome sequences for each paired sample for both the remdesivir and control groups were aligned using MAFFT v7.453 software and an ML phylogeny was inferred with IQ-TREE v2.0.5 using the SARS-CoV-2 reference genome Wuhan-Hu-1 (GenBank MN908947.3) to root the tree (as in ref. 70) with its ModelFinder function (71) to estimate the nucleotide substitution model best fitted by means of Bayesian information criterion (BIC). The best-fitted model was GTR+F+R3, and we assessed the tree topology for the phylogeny both with the Shimodaira–Hasegawa approximate likelihood-ratio test (SH-aLRT) (72) and with ultrafast bootstrap (UFboot) (73), with 1,000 replicates each. Hamming distance (number of nucleotide substitutions) was calculated for each pair of consensus sequences from the same patient using MEGA X (74) to estimate viral divergence before and after remdesivir administration or hospitalization. To test for significant changes in divergence between remdesivir and control, we fitted a linear model assuming a negative binomial distribution of the Hamming distance and controlling for the time between samples using the R package glmmTMB 1.1.8.

### Viral diversity analysis.

To study and compare intra-host diversification, we calculated Shannon entropy using the nucleotide frequencies per position relative to the SARS-CoV-2 reference genome (Wuhan-Hu-1). Nucleotide frequencies were obtained from deep-sequencing read data by iVar using the “variants” function and a frequency threshold greater than 3% (75). Shannon entropy was calculated using the formula [*Sh* = *SUM*[–(*pi*) * log_2_(*pi*)], where *Sh* is Shannon entropy calculated for each position and *pi* is the frequency of each nucleotide in each position. To ensure a robust estimation of diversity, Shannon entropy calculations were limited to positions with a mutation frequency significantly higher than the mean error rate at each position using a Fisher’s exact test *P* < 0.05 calculated by iVar. Overall genetic entropy in a specimen was calculated as the sum of entropy values for each nucleotide position across the genome; genetic entropy in a viral gene was calculated as the sum of entropy values for each nucleotide position in each gene. Note that insertions and deletions were not considered in these analyses.

To test for differences in overall entropy between “pre” and “post” specimens in each cohort, we used a linear mixed-effects model controlling for time between paired specimens and within-patient variability. Likewise, we tested for changes in Shannon entropy between pre and post per gene within each cohort by fitting a linear mixed effects model while controlling for time between samples and within-patient variability. *P* values were adjusted for FDR using the Benjamini-Hochberg method. Finally, changes in Shannon entropy per position were tested for positions that showed *Sh* > 0 at least in 1 of the 2 paired samples for 4 or more patients (156 positions were tested for the remdesivir cohort and 56 for the control cohort). A linear mixed-effects model was fitted for each of these positions while controlling for time between samples, viral load, and within-patient variability. All obtained *P* values were subsequently adjusted for FDR to control for multiple comparisons. Due to the high number of positions tested, positions with an adjusted *P* value less than 0.1 were considered significant.

For these analyses we used lme4 version 1.1–34 in R version 4.0.3. Wilcoxon’s and Mann-Whitney *U* analyses were done in Python (v3.8.8) using the Pandas (v1.1.5), NumPy (v1.23.2), and SciPy (v1.6.2) packages. All graphs were generated in Matplotlib (76) (v3.3.4) and seaborn (77) (v0.11.1).

### Literature review.

In the systematic review, a computerized search was implemented in PubMed using search term (“SARS-CoV-2” OR “Coronavirus”) AND (“Remdesivir” OR “antiviral”) AND (“polymerase” OR “rna-dependent” OR “nsp12”) AND (“resistance” OR “mutation” OR “mutagenesis” OR “variant” OR “persistent”). This search was limited to publication dates between 2020 and 2024 was completed through April 5, 2024. This resulted in a total of 265 articles. In addition, we reviewed the reference sections of relevant articles. We included studies that identified at least 1 mutation related to resistance in remdesivir. We excluded other meta-analyses as well as review articles. Records were manually screened for relevance. This resulted in a total of 19 relevant articles.

### Structural visualization of putative remdesivir-resistant mutations.

Remdesivir-bound (PDB 6NUS) and apo (PDB 7BV2) nsp12 x-ray crystal structures were visualized using PyMOL v. 2.4.1. Nsp12 in vivo, in vitro, and in silico mutations were annotated as surface representations on the apo crystal structure. Likewise, amino acid residue side chains within 6 Å of the remdesivir binding site were individually annotated and visualized on the remdesivir-bound structure.

### Data availability.

All of the code, tools, and parameter settings required to reproduce these results, including our genome assembly pipeline and within-host variant analysis, are available on GitHub at https://github.com/NU-CPGME/rdv_resistance/commit/cd7c987f720074cf647734a3a828007601f3d513 All viral consensus sequences have been uploaded to GISAID (GISAID accession numbers provided in Supplemental Table 2). The raw reads from each sequencing run have been deposited to the NCBI Sequence Read Archive (SRA accession numbers provided in Supplemental Table 2) and have been uploaded as BioProject PRJNA1177876 (BioSample accession numbers provided in Supplemental Table 2). The individualized clinical data reported in this study cannot be deposited in a public repository due to IRB constraints, but population-level metrics are provided in Tables 1 and 2, and Ct values are reported in the Supporting Data Values file.

## Supplementary Material

Supplemental data

Supplemental table 1

Supplemental table 2

Supplemental table 3

Supporting data values

## Figures and Tables

**Table 1:**
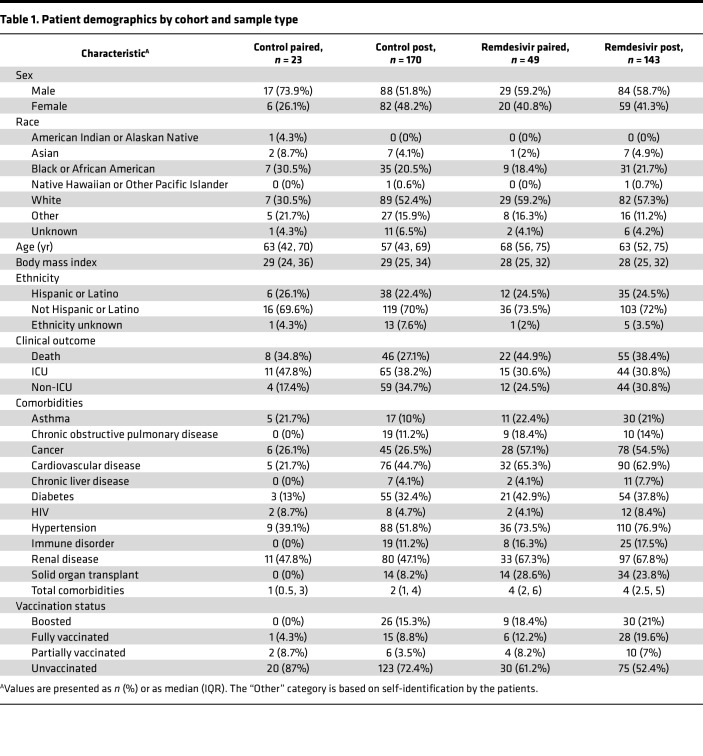
Patient demographics by cohort and sample type

**Table 2:**
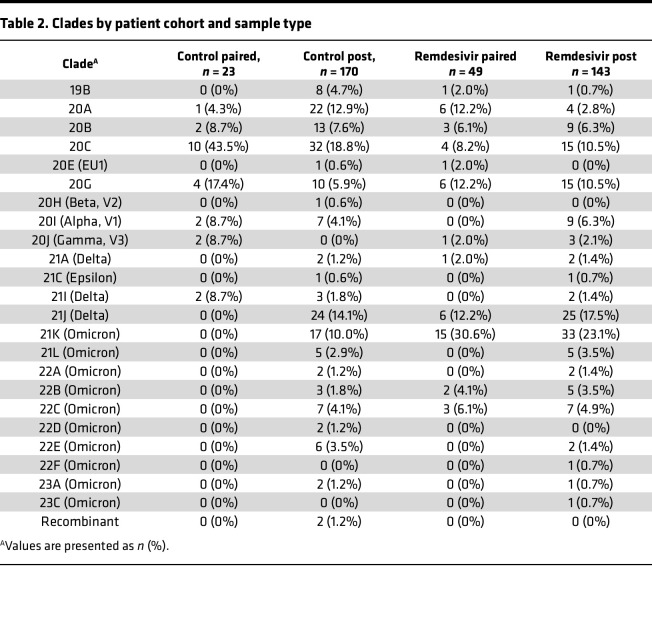
Clades by patient cohort and sample type

## Version 1 01/21/2025

In-Press Preview

## Version 2 02/24/2025

Electronic publication
