# Supplementary material for: The impact of remdesivir on SARS-CoV-2 evolution in vivo
Source: JCI Insight. 2025 Jan 21;10(4):e182376. doi: 10.1172/jci.insight.182376 (PMC11949014; doi:10.1172/jci.insight.182376)
Supplement: Supplemental data [file jciinsight-10-182376-s102.pdf]

SUPPLEMENTARY INFORMATION

for

The Impact of Remdesivir on SARS-CoV-2 Evolution *in vivo*

Ted Ling-Hu[1,2], Lacy M. Simons[1,2], Estefany Rios-Guzman[1,2], Alexandre Machado de Sant'Anna Carvalho[1,2] ,Maria Francesca Agnes[1,2], Arghavan Alisoltanidehkordi[1,2], Egon A. Ozer[1,2], Ramon Lorenzo-Redondo[1,2,+], Judd F. Hultquist[1,2,+]

[1] Division of Infectious Diseases, Northwestern University Feinberg School of Medicine, Chicago, IL 60611, USA.

[2] Center for Pathogen Genomics and Microbial Evolution, Northwestern University Havey Institute for Global Health, Chicago, IL 60611, USA.

[+] Corresponding authors: Ramon Lorenzo-Redondo, Ph.D. (ramon.lorenzo@northwestern.edu); Judd F. Hultquist, Ph.D. (judd.hultquist@northwestern.edu)

TABLE OF CONTENTS

Supplemental Figure 1……………………………………pages 2-3

Supplemental Figure 2……………………………………pages 4-5

Supplemental Table Legends…………………………….page 6

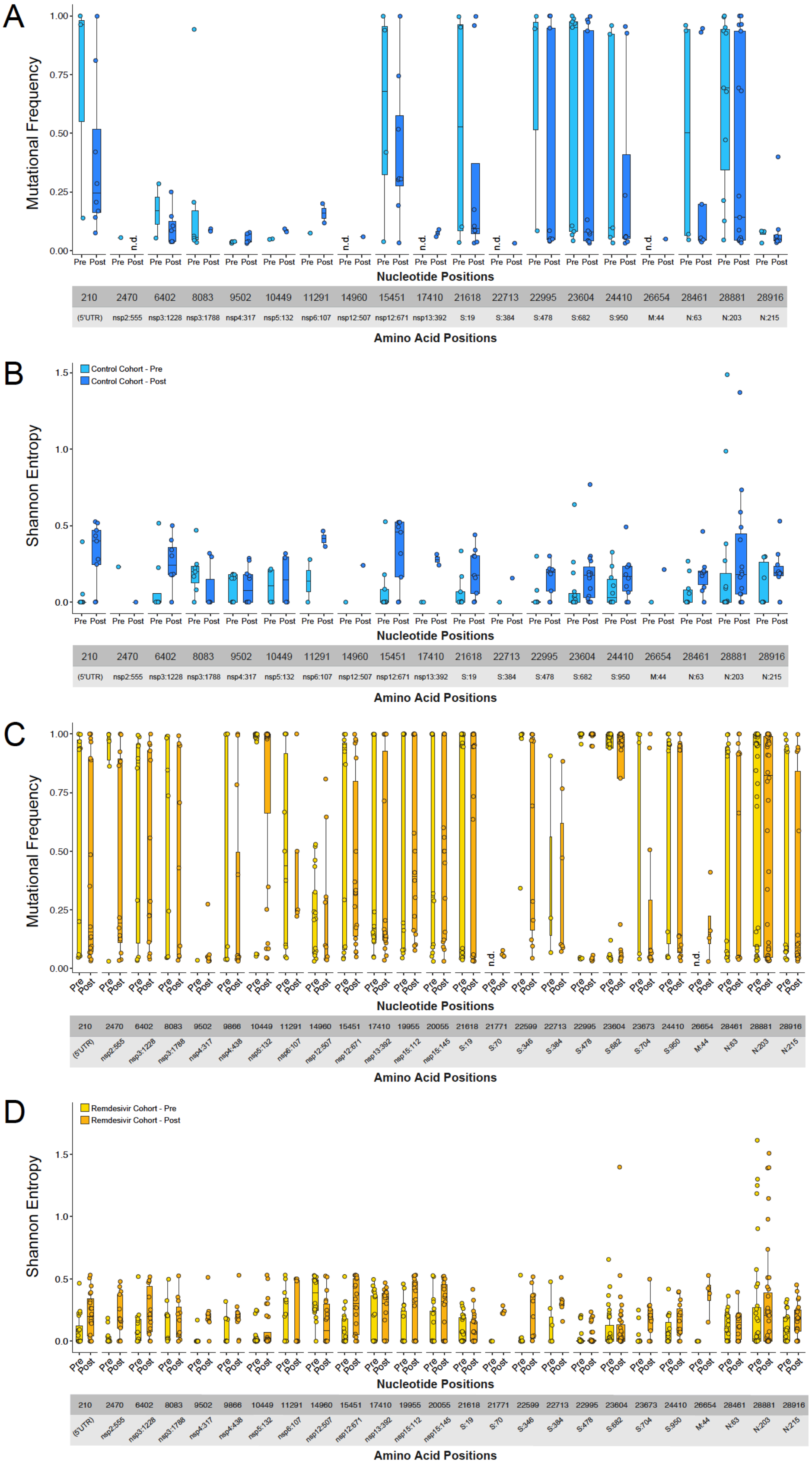
A
Mutational Frequency
Nucleotide Positions
210 2470 6402 8083 9502 10449 11291 14960 15451 17410 21618 22713 22995 23604 24410 26654 28461 28881 28916
(5'UTR) nsp2:555 nsp3:1228 nsp3:1788 nsp4:317 nsp5:132 nsp6:107 nsp12:507 nsp12:671 nsp13:392 S:19 S:384 S:478 S:682 S:950 M:44 N:63 N:203 N:215
Amino Acid Positions
B
Control Cohort - Pre
Control Cohort - Post
Shannon Entropy
Nucleotide Positions
210 2470 6402 8083 9502 10449 11291 14960 15451 17410 21618 22713 22995 23604 24410 26654 28461 28881 28916
(5'UTR) nsp2:555 nsp3:1228 nsp3:1788 nsp4:317 nsp5:132 nsp6:107 nsp12:507 nsp12:671 nsp13:392 S:19 S:384 S:478 S:682 S:950 M:44 N:63 N:203 N:215
Amino Acid Positions
C
Mutational Frequency
Nucleotide Positions
210 2470 6402 8083 9502 9866 10449 11291 14960 15451 17410 19955 20055 21618 21771 22599 22713 22995 23604 23673 24410 26654 28461 28881 28916
(5'UTR) nsp2:555 nsp3:1228 nsp3:1788 nsp4:317 nsp4:438 nsp5:132 nsp6:107 nsp12:507 nsp12:671 nsp13:392 nsp15:112 nsp15:145 S:19 S:70 S:346 S:384 S:478 S:682 S:704 S:950 M:44 N:63 N:203 N:215
Amino Acid Positions
D
Remdesivir Cohort - Pre
Remdesivir Cohort - Post
Shannon Entropy
Nucleotide Positions
210 2470 6402 8083 9502 9866 10449 11291 14960 15451 17410 19955 20055 21618 21771 22599 22713 22995 23604 23673 24410 26654 28461 28881 28916
(5'UTR) nsp2:555 nsp3:1228 nsp3:1788 nsp4:317 nsp4:438 nsp5:132 nsp6:107 nsp12:507 nsp12:671 nsp13:392 nsp15:112 nsp15:145 S:19 S:70 S:346 S:384 S:478 S:682 S:704 S:950 M:44 N:63 N:203 N:215
Amino Acid Positions

Supplemental Figure 1 | Mutational frequency and Shannon entropy at sites of diversification or purification over time. **A)** Mutational frequency at the indicated positions from **Figure 4B** in the SARS-CoV-2 genome relative to the Wuhan-Hu-1 reference genome (accession number: MN908947.3) in “pre” and “post” specimens from the control cohort. Nucleotide and amino acid positions are provided below. Only specimens with >1% mutational frequency at a given position are shown (positions that were not detected listed as “n.d.”). Boxplots represent the median (center line) and first/third quartiles (box), with tails extending 1.5 times the interquartile range (IQR). **B)** Shannon entropy $[Sh = SUM[-(pi) * log2(pi)]$, where pi is nucleotide frequency] in “pre” and “post” specimens from the control cohort at the positions indicated in A. Boxplots represent the median (center line) and first/third quartiles (box), with tails extending 1.5 times the interquartile range (IQR). **C)** Mutational frequency and **D)** Shannon entropy at the indicated positions from **Figure 4B** in “pre” and “post” specimens from the remdesivir cohort.

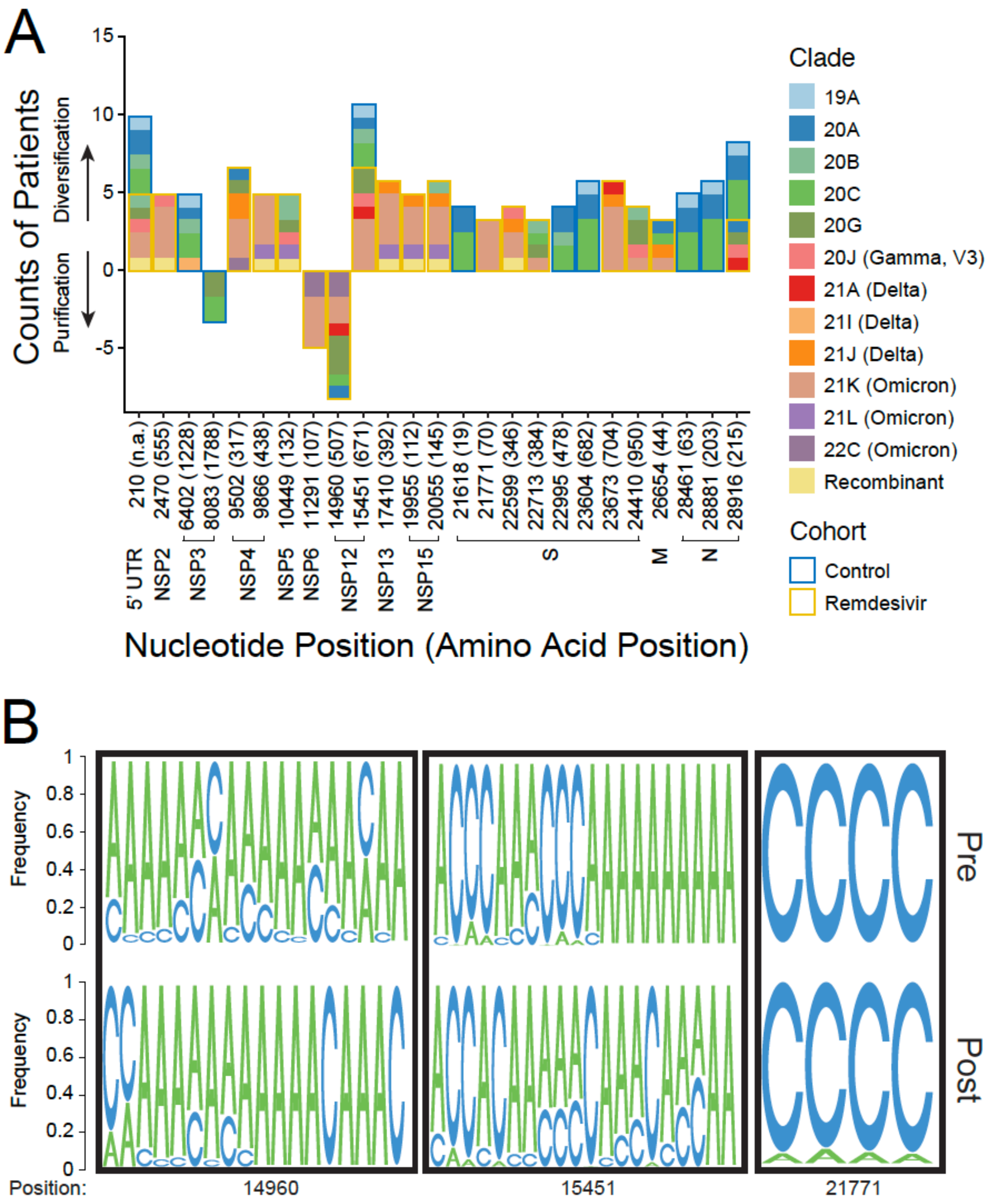

A
Counts of Patients
Diversification
Purification
Clade
19A
20A
20B
20C
20G
20J (Gamma, V3)
21A (Delta)
21I (Delta)
21J (Delta)
21K (Omicron)
21L (Omicron)
22C (Omicron)
Recombinant
Cohort
Control
Remdesivir
210 (n.a.)
2470 (555)
6402 (1228)
8083 (1788)
9502 (317)
9866 (438)
10449 (132)
11291 (107)
14960 (507)
15451 (671)
17410 (392)
19955 (112)
20055 (145)
21618 (19)
21771 (70)
22599 (346)
22713 (384)
22995 (478)
23604 (682)
23673 (704)
24410 (950)
26654 (44)
28461 (63)
28881 (203)
28916 (215)
5' UTR
NSP2
NSP3
NSP4
NSP5
NSP6
NSP12
NSP13
NSP15
S
M
N
Nucleotide Position (Amino Acid Position)
B
Frequency
Pre
Post
Position:
14960
15451
21771

Supplemental Figure 2 | Clade distribution and nucleotide frequencies of selected positions following remdesivir treatment. **A)** Stacked bar plot illustrating the number of patients in each cohort that exhibit change in Shannon entropy (diversity) at a given position in paired specimens. Shannon entropy was calculated as $Sh = SUM[-(pi) * log2(pi)]$, where pi is nucleotide frequency. Positions where at minimum four patients in either cohort showed increase in Shannon entropy from zero (*i.e.*, diversification) or decrease to zero (*i.e.*, purification) are shown. Cohort membership is indicated by the blue (control) and yellow (remdesivir) outlines. The colors inside the bar chart refer to the clade of the virus in which the diversification or purification event was observed. **B)** Sequence logo plots depicting nucleotide frequency at the three nucleotide positions (14960, 15451, and 21771) that had significant changes following remdesivir administration in each set of paired patient isolates for which some mutational frequency was observed either in the ”pre” or “post” specimen.

SUPPLEMENTAL TABLES

Supplemental Table 1 | Summary of previously identified remdesivir resistance mutations and their respective references.

Supplemental Table 2 | Summary of isolates analyzed in this study including clade designations, lineage designations, GISAID accession numbers, and NCBI accession numbers.

Supplemental Table 3 | Summary of consensus level mutations found between matched “pre” and “post” specimens in the control and remdesivir cohorts.
